# Host, pathogen and environment: a bacterial gbpA gene expression study in response to magnesium environment and presence of prawn carapace and commercial chitin

**DOI:** 10.1186/s13099-016-0105-5

**Published:** 2016-05-26

**Authors:** Suma Tiruvayipati, Subha Bhassu

**Affiliations:** Department of Genetics and Molecular Biology, Institute of Biological Sciences, Faculty of Science, University of Malaya, 50603 Kuala Lumpur, Malaysia; Center of Biotechnology for Agriculture (CEBAR), University of Malaya, Kuala Lumpur, Malaysia

**Keywords:** *Vibrio parahaemolyticus*, *Macrobrachium rosenbergii*, MgSO_4_·7H_2_O, Carapace, Commercial chitin flakes

## Abstract

**Background:**

*Vibrio parahaemolyticus* is a Gram-negative halophilic bacterium which is found largely in estuarine and coastal waters. The bacteria has been a main focus in gastro-intestinal infections caused primarily due to the consumption of contaminated seafood. It was shown to survive in magnesium concentrations as high as 300 mM which are toxic to various other micro-organisms. Several genes of *V. parahaemolyticus* were studied, among which gbpA (N-acetyl glucosamine binding protein) was reported in *Vibrio cholerae*.

**Methods:**

The current study investigates the *V. parahaemolyticus* gbpA gene expression at different concentrations of magnesium sulfate heptahydrate (MgSO_4_·7H_2_O, chosen as the magnesium environment), in the presence of the host’s (prawn) carapace and the mimicked carapace [commercial chitin flakes (Sigma)]. The concentrations of MgSO_4_·7H_2_O utilized were approximately 0, 1, 75, 137, 225 and 300 mM. These were selected based upon the survival conditions required by prawn and bacteria, respectively. 0.05 gm/3 ml of carapace (by dry weight) and commercial chitin flakes were used in the experiments. Bacterial count was performed for the biological triplicates for the 3 experimental setups. The genome of *Vibrio parahaemolyticus* PCV08-7 (VPPCV08-7) was used as a reference, based on whose translated gbpA gene the probable protein-chemical interactions were determined on the STITCH database.

**Results:**

The GbpA protein was shown to interact with chitin on the STITCH database. In our experiments, the gbpA showed lower gene expression levels at different MgSO_4_·7H_2_O concentrations in the presence of chitin and carapace, than with the presence of only MgSO_4_•7H_2_O. In addition, the bacterial count for various concentrations of magnesium used, revealed a distinct decrease in bacterial count within and among each of the three experimental setups.

**Conclusion:**

In the presence of only magnesium, an increase in the gbpA expression with neither chitin nor carapace and vice versa supported by the results from the bacterial counts could help further studies to prove that the moulting phase of prawns may trigger increased expression of the *V. parahaemolyticus* gbpA gene.

**Electronic supplementary material:**

The online version of this article (doi:10.1186/s13099-016-0105-5) contains supplementary material, which is available to authorized users.

## Background

*Vibrio parahaemolyticus* is a curved, rod-shaped Gram-negative bacterium. It is non-spore forming and has a high motility rate due to its polar flagellum. Through a mechanism known as swarming, these microorganisms migrate across semi-solid surfaces [1] with the help of their lateral flagella. Across the world, inshore marine waters are densely populated with *V. parahaemolyticus* which is particularly common in estuarine marine water. Research has shown that *V. parahaemolyticus* is seasonal [2] and thrives well in warmer conditions. For example, the bacteria could not be detected during the winter (November–March) in Chesapeake Bay seawater [2]. On the other hand, *V. parahaemolyticus* begins to multiply when there is an increase in temperature [2]. This could be a result of the microorganism somehow being reintroduced into the sea water or its emergence from marine sediments in which it could have survived throughout the winter.

Temperatures ranging from 35 to 39 °C [3] are the optimal conditions for the growth of *V. parahaemolyticus*. It has a generation time of less than 20 min, although it can double in as little as 5 min [4] under certain conditions. As a result, *V. parahaemolyticus* is most commonly observed in the warm season as a mesophilic bacterium causing food-borne outbreaks which peak in summer [5, 6], the levels of *V. parahaemolyticus* found in freshly harvested seafood tend to be rather lower than the predicted infection doses [7]. However, the ability of the bacterium to multiply very rapidly at suitable temperatures means that its presence in food is often enough to cause disease.

*Vibrio parahaemolyticus* has one very important requirement to live and multiply that is salinity. *V. parahaemolyticus* typically encounters salinity concentrations in the marine environment ranging between 0.8 and 3 % [8]. It requires optimal salinity levels between 1 and 3 %, but laboratory studies have shown that *V. parahaemolyticus* can thrive in between 0.5 and 10 % sodium chloride concentrations.

*Vibrio parahaemolyticus* isolates were found to survive even in 300 mM magnesium (e.g. in severely polluted coastal waters in some parts of India)—a level considered toxic to many other microorganisms [9]. It’s survival under such wide-ranging conditions may be due to its ability to utilize magnesium. A 5.5 kb plasmid is said to carry the genes responsible for the bacterium’s high resistance to high magnesium concentrations [9]. Injured or thermally treated *V. parahaemolyticus* cells display an increased uptake of magnesium suggesting a possible increased requirement for magnesium not only for the stability and repair [10] of the ribosome, but also of the cell membrane. To sum up, *V. parahaemolyticus*’s ability to survive in high concentrations of magnesium or other metal ions allows it to out-compete other basic seawater flora in terms of survival and growth in such drastic environmental conditions.

The giant freshwater prawn *Macrobrachium rosenbergii* is a freshwater aquatic organism. The optimal temperature range for *Macrobrachium rosenbergii* larvae to survive is 28 to 31 °C. Observations have shown that a salinity of <10 parts per thousand (ppt) is ideal for freshwater prawn hatcheries (http://www.fao.org/docrep/005/y4100e/y4100e04.htm#P193_35649). While calcium is important for the formation of the prawn exoskeleton (http://www.thefishsite.com/articles/464/moulting-and-behaviour-changes-in-freshwater-prawn), the crucial element for this species is a favourable condition for the survival of its larvae.

Various reports have suggested that magnesium is an important component of the environment for prawn survival particularly for juvenile prawns [11]. A recent article [12] describing the effects of salinity through the use of artificial sea water clearly explains the importance of magnesium in the survival amounts of post larvae. Taking an example, the effect of an environment that is acidic due to the presence of aluminium could not hinder the survival stages of post larvae due to the presence of increased levels of magnesium ions (Mg^2+^) [13]. The characteristics of water which are good for prawn hatcheries are said to be 10–27 parts per million (ppm) of magnesium in fresh water, 1250–1345 ppm Mg in seawater and 460–540 ppm Mg in brackish water (http://www.fao.org/docrep/005/y4100e/y4100e04.htm#P193_35649). These features and conditions show how important magnesium ion is for the survival of larvae which undergo a very critical moulting stage before reaching the post-larval stage.

Most *Vibrio* species have adapted to aquatic organisms and caused severe infections on consumption by humans. *V. parahaemolyticus* has several virulence, pathogenicity and antibiotic resistance factors which show that it can survive well in aquatic organisms, especially the giant freshwater prawn, *M. rosenbergii* [14].

Detailed studies of the growth conditions of *M. rosenbergii* in the environment can help us to correlate the respective levels of adaptability of *V. parahaemolyticus* to *M. rosenbergii*. Studies have shown that *M. rosenbergii* can survive in a range of different media compositions with varying proportions of NaCl, KCl and MgCl_2_ + MgSO_4_ [5]. However, the fertilization envelope of shrimp eggs was observed to grow thin when there is a depletion of calcium and magnesium [15]. Early-stage embryos were shown to require optimal levels of medium containing MgCl_2_ + MgSO_4_ for their proper development [16].

The role of magnesium ion in the normal hatching rate of larvae has not been shown to be significant [16]. However, the importance of magnesium in survival mechanisms was observed [12] as explained earlier. Perhaps the most interesting similarity of *V. parahaemolyticus* to prawn is its unusually good tolerance levels to high concentrations of magnesium and its growth capability under iron-limiting conditions—both of which are quite a match to the conditions of prawn larvae survival.

In addition, another important factor is the N-acetyl glucosamine binding protein (GbpA) reported in *Vibrio cholerae* [17, 18] to have the property to bind to epithelial cell surfaces and chitin of the host surface. An in vitro study in 1996 presented how cell associated N-acetyl D-glucosamine specific haemagglutinin of *Vibrio cholerae* O1 showed adhesive characteristics to the rabbit intestinal epithelial cells [19]. In 2008, gbpA gene of *V. cholerae* was studied in specific with mucin for its co-operative levels of gene expression ultimately giving way to intestinal colonization and infection by the bacterium [20]. In infant mouse models it was observed that a deletion in the adhesion gbpA portrayed a deficit in the intestinal colonization [21, 22]. The importance of gbpA in the intestinal colonization of *V. cholerae* was reported by a study along with several other colonizing factors [23]. Our study aims at checking the levels of bacterial gbpA gene expression in the presence of the host carapace and commercial chitin at different magnesium environment concentrations. This study could help researchers to consider environment as an indispensable factor in host-pathogen studies, not only in seafood industries, but even in omics studies.

## Methods

### Protein-chemical interactions

The STITCH v1.9 [24] database for protein-chemical interactions was used to check the interactions of the gbpA gene of the VPPCV08-7 [25] with other proteins molecules on the database.

### *Vibrio parahaemolyticus* PCV08-7 culture conditions

The inocula were first prepared by using the VPPCV08-7 glycerol stocks [25]. The glycerol stock was used to revive the bacteria in 5 ml Luria-bertani broth (LB broth) with 2 % NaCl as a primary culture incubated overnight at 37 °C in a shaking incubator at 220 rpm. 30 ml LB broth with 2 % NaCl was then inoculated with 5 % primary culture containing cells at the mid-exponential phase. This inocula was further used for all the 3 experimental setups. Eighteen, 10 ml falcon tubes (Greiner bio-one, North America) were used under aseptic conditions to pour LB broth with 2 % NaCl in each tube and then inoculated with 5 % of the prepared inocula to make up to 6 ml of the inoculated culture. These 18 culture tubes contained three sets of experiments as follows with six concentrations of magnesium sulphate hepta hydrate (MgSO_4_·7H_2_O) (chosen as the magnesium environment in the study) used (stock prepared was 500 mM). These concentrations of MgSO_4_·7H_2_O correspond to 0 ppm (0 mM), 300 ppm (1 mM), 18,500 ppm (75 mM), 34,000 ppm (137 mM), 55,500 ppm (225 mM) and 73,941 ppm (300 mM), respectively per set. The first set consisted of these six concentrations of MgSO_4_·7H_2_O. 0.05 g/3 ml by dry weight of carapace of the prawn was added per tube to all six concentrations of the second set, while 0.05 g/3 ml of commercial chitin flakes (chitin from shrimp shells, SIGMA) was added to the third set. All the 18 experiments were carried out in triplicates at 37 °C in a shaking incubator at 220 rpm. For the isolation of total RNA all the 18 bacterial cultures (3 ml) were harvested at the 16th hour (for maximal turbidity/growth).

### VPPCV08-7 bacterial plate count

One milliliter each of all the 18 experiments in triplicates was centrifuged at 8000 rpm at 4 °C for 2 min and the pellet was dissolved in 750 μl of fresh LB broth (2 % NaCl) and 5 μl of each was spread plated on thiosulfate-citrate-bile-sucrose (Difco, France) agar plates. After incubation at 37 °C for 24 h, the bacterial plate count was performed (Additional file 1: Figure S1).

### Total RNA isolation and reverse transcription (RT) PCR

Total RNA was isolated from the triplicates of the three experimental setups above using a Promega Total RNA isolation kit and converted to cDNA using a reverse transcription PCR protocol (Additional file 1: Table S1). 4 µl of each isolated RNA sample from each of the triplicates was added to 1 µl Random primer to make a reaction mixture of 5 µl for an initial incubation at 70 °C/5 min, followed by 4 °C/5 min in a Biorad PCR machine. A reverse transcription mix of 15 µl each (6.1 µl Nuclease free water, 4 µl of 5× reaction buffer, 2.4 µl MgCl_2_, 1 µl dNTPs, 0.5 µl ribonuclease inhibitor and 1 μl reverse transcriptase) was added to the former mixture for annealing at 25 °C/5 min, extension at 42 °C/60 min, and heat inactivation of reverse transcriptase at 70 °C/15 min.

### Quantitative Real Time (qRT)—PCR

We selected the gbpA gene as identified from the STITCH v1.9 protein-chemical interactions for qRT-PCR to check the effects of MgSO4. 7H2O as the environment in the absence and presence of commercial chitin and carapace. The bacterial house-keeping gene RNA helicase (deaD) was selected as the internal control gene for qRT-PCR to later normalize the amounts of RNA. The tools used for primer designing were AmliFX, DNA star [26], Primer3 (http://www.bioinformatics.nl/cgi-bin/primer3plus/primer3plus.cgi/) and NCBI primer Blast (http://www.ncbi.nlm.nih.gov/tools/primer-blast/). The primers were specifically designed considering the domain regions of deaD (product size 181 bp corresponds to DeaD-box helicases) and gbpA (product size 190 bp corresponds to chitin binding domain) as follows: deaD forward primer 5′-GTGCACACGTTGTTGTTGGT-3′, reverse primer 5′-AGAACGCGTTGTGCTGATTC-3′ and gbpA forward primer 5′-CTCGTTCGCTCTCAACCCTT-3′, reverse primer 5′-CACAGGGTCGTCACCATCAA-3′. The qRT-PCR reaction (20 μl) consisted of 10 µl Power SYBR green PCR master mix, 0.6 µl forward primer, 0.6 µl reverse primer, 1 µl DNA template and 7.8 µl ultrapure water. The default thermal cycling conditions were used for the run with stage 1 at 50 °C/2 min, stage 2 at 95 °C/10 min for one cycle, stage 3 with 40 repetitions at 95 °C/15 s, followed by 60° C/1 min, carried out by using the Applied Biosystems 7500 Real Time PCR system. Similar qRT-PCR profile was applied to the internal control gene, deaD. The gene expression levels of the 3 experimental setups were analysed by using the comparative 2^−ΔΔCT^ method {2[−Delta Delta C(T)] Method} [27] known as Livak method.

## Results and discussion

### Bioinformatic analysis of host-pathogen genes

**Fig. 1:**
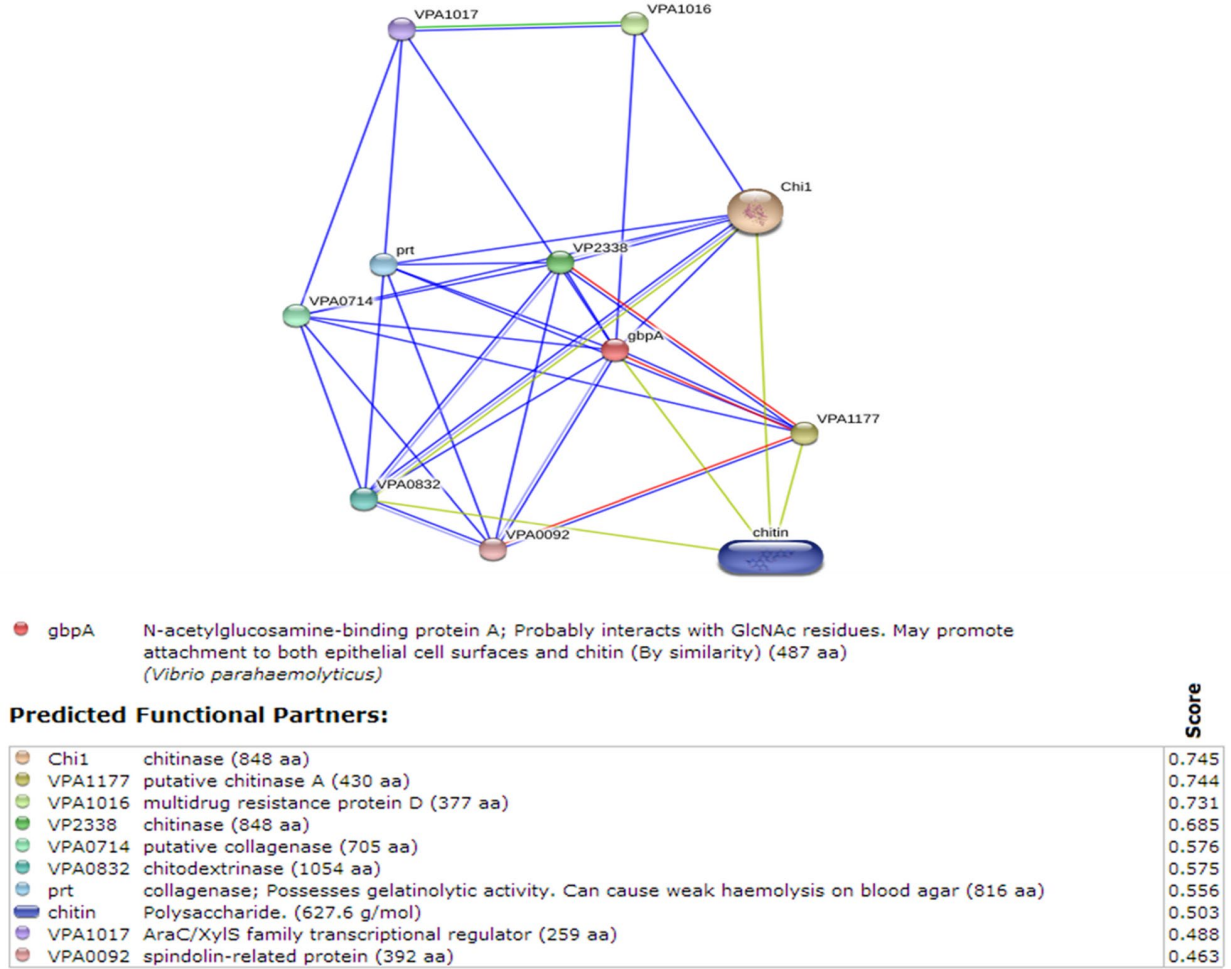
STITCH 3 protein-chemical interactions of gbpA protein of *Vibrio parahaemolyticus.* Protein–chemical interaction tests of gbpA (*N*-acetylglucosamine-binding protein A) on the STITCH 3 database show interactions primarily with genes of *Vibrio parahaemolyticus* (VPA1016-multidrug resistance protein D, VPA1017-AraC/XylS family transcriptional regulator, VP2338-chitinase, VPA1177-putative chitinase A, VPA0092-spindolin-related protein, VPA0832-chitodextrinase, VPA0714-putative collagenase, prt-collagenase, Chi1–chitinase, and chitin) (*Source*: Tiruvayipati S, Bhassu S: Host, pathogen and the environment: the case of *Macrobrachium rosenbergii, Vibrio parahaemolyticus and magnesium.* Gut Pathog 2016, 8:15)

Previous studies have shown that the gene gbpA of *V. cholerae* interacts with the intestinal epithelial chitinous membrane or host surfaces [18, 20]. As *Macrobrachium rosenbergii* is known to be infected by *Vibrio parahaemolyticus,* we selected this gene to check for interactions with other proteins or chemicals as bacterial chitin-binding proteins were previously shown to be virulent [28]. The protein-chemical interactions of *Vibrio parahaemolyticus* gbpA revealed relationships with a multidrug resistance protein D (VPA1016), AraC/XylS family transcriptional regulator (VPA1017), chitinase (VP2338), putative chitinase A (VPA1177), spindolin-related protein (VPA0092), chitodextrinase (VPA0832), putative collagenase (VPA0714), prt collagenase, Chi1–chitinase, and chitin. Fig. 1 obtained from STITCH 3, shows the predicted interaction with chitin, an epithelial cell surface component and a major component of the prawn shell. This result helped us select chitin for our study in accordance with the earlier explained gbpA gene interaction with the epithelial chitinous membrane [20].

### *Vibrio parahaemolyticus* gbpA gene expression study in response to magnesium and carapace/commercial chitin

**Fig. 2:**
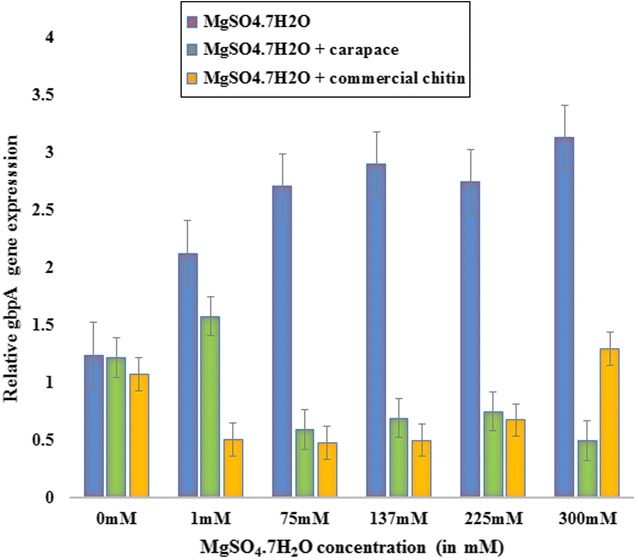
*Graph* representing relative gbpA gene expression in terms of 2^−ΔΔCT^ values in the presence of different MgSO_4_·7H_2_O concentrations, MgSO_4_·7H_2_O with commercial chitin and MgSO_4_·7H_2_O with carapace

The various concentrations of MgSO_4_·7H_2_O were selected based on the previous literature which studied the levels of magnesium required for the survival of prawn [12] as well as for *V. parahaemolyticus* [9]. The lowest and the highest ppm values for the concentrations of MgSO_4_·7H_2_O were selected based on the same as previously, no such research was reported on this aspect of *V. parahaemolyticus* gene expression study. The relative gbpA gene expression levels were calculated using the livak method [27]. The house-keeping gene deaD expression was used for qPCR normalization with the target gene being gbpA. With increasing concentrations of MgSO_4_·7H_2_O a uniform increase in the gbpA gene expression was observed (Fig. 2). In the presence of commercial chitin, the level of gbpA gene expression was high at 0 mM MgSO_4_·7H_2_O, but then a gradual increase in gene expression was observed with increase in concentration of MgSO_4_·7H_2_O. Lastly, in the presence of carapace the levels of gbpA gene expression increased at 0 and 1 mM MgSO_4_·7H_2_O after which the gene expression levels dropped greatly at the 75 mM MgSO_4_·7H_2_O followed by a steady increase and a final drop at the 300 mM MgSO_4_·7H_2_O. We therefore infer that in all three experimental setups similar levels of gbpA gene expression were observed at 0 ppm MgSO_4_·7H_2_O with a comparatively slightly lower gene expression in the presence of chitin. This explains that the bacterial gbpA expression could have a miniature dependency on the presence of carapace or chitin in the environment, but this is subject to further validation with fluctuating the amount of carapace/chitin used in future studies. As our research is primarily to check the effect of an external environment, we were successfully able to identify that the increase in gbpA gene expression directly depends on the increasing concentrations of MgSO_4_·7H_2_O. Our result backed by further research could support the *V. parahaemolyticus* survival in high magnesium concentrations as well as its affinity to 0.8 to 3 % salinity range [8, 9].

The results of the three experiments were quite significant in the *V. parahaemolyticus* culture treated with only MgSO_4_·7H_2_O with a *P* value of 0.04, with both MgSO_4_·7H_2_O and chitin present with a P-value of 0.000441 and with MgSO_4_·7H_2_O and carapace present with a P-value of 0.0152 (Additional file 1: Table S2–S4). Concentrations of 300 mM (P-value = 0.02744) and 75 mM (P-value = 0.00132) produced highly significant values in the samples treated with MgSO_4_·7H_2_O and MgSO_4_·7H_2_O with chitin, respectively (Additional file 1: Table S5).

### *Vibrio parahaemolyticus* bacterial count in response to magnesium & carapace/commercial chitin

**Fig. 3:**
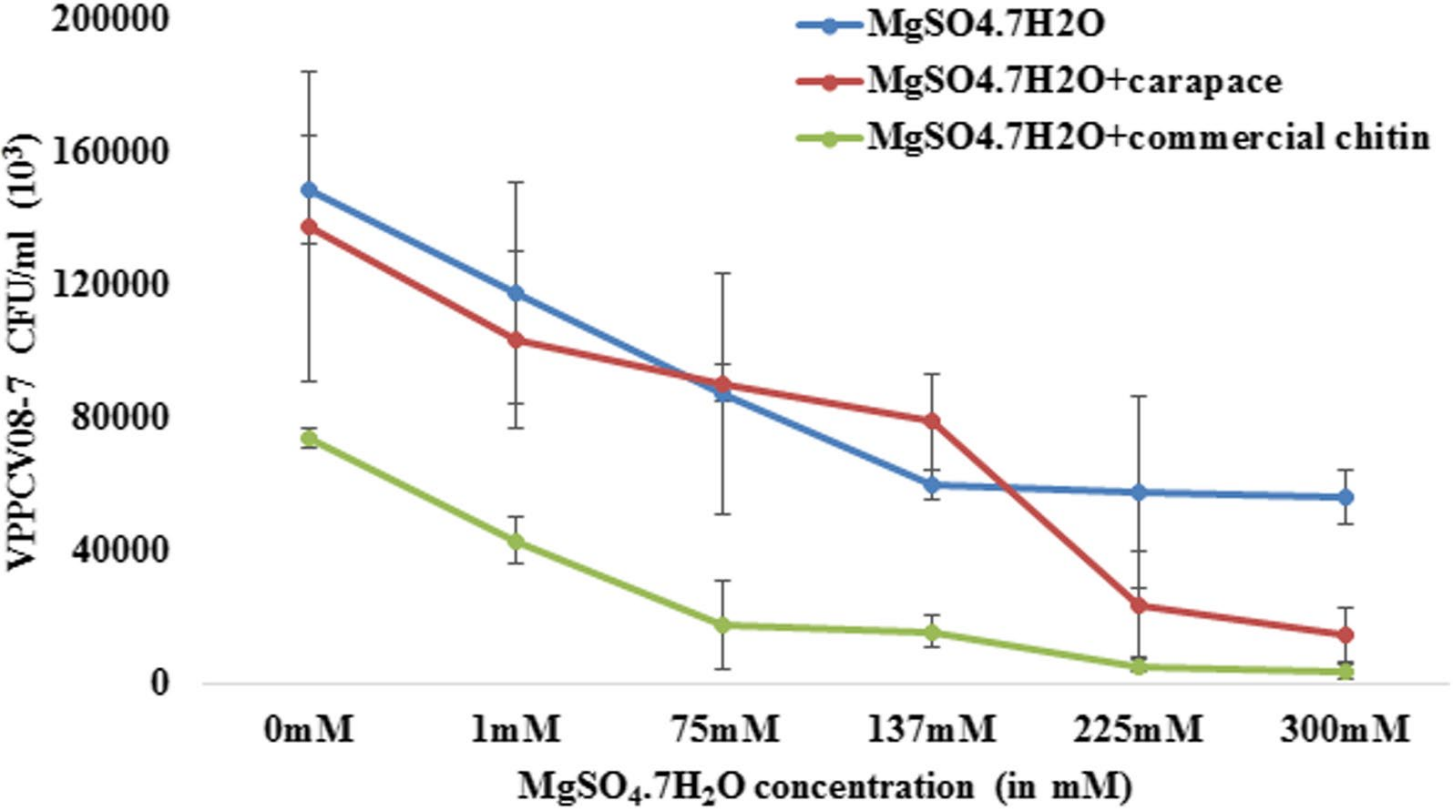
Graphical representation of *Vibrio parahaemolyticus* PCV08-7 CFU/ml in the three experimental setups: in the presence of different MgSO_4_·7H_2_O concentrations, MgSO_4_·7H_2_O with commercial chitin and MgSO_4_·7H_2_O with carapace

**Fig. 4:**
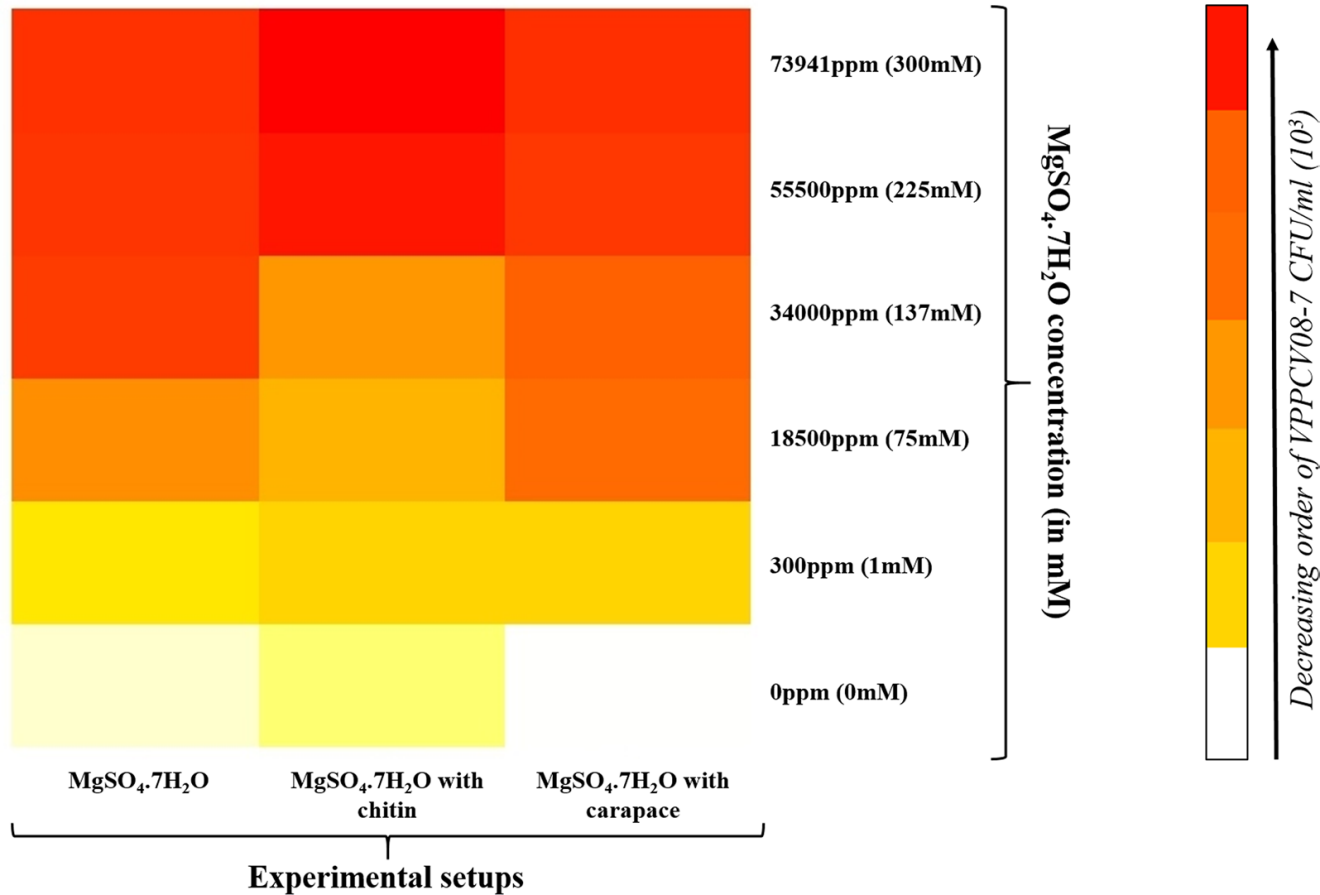
Heat map of decreasing order of *Vibrio parahaemolyticus* PCV08-7 CFU/ml in the three experimental setups at different MgSO_4_·7H_2_O concentrations

The colony forming units (CFU) per millilitre (ml) of VPPCV08-7 in the presence of MgSO_4_·7H_2_O, MgSO_4_·7H_2_O with chitin, and MgSO_4_·7H_2_O with carapace, showed a significant decrease in all three experimental setups with increase in concentrations of MgSO_4_·7H_2_O (Table 1). This data supports that though the *V. parahaemolyticus* growth is affected due to the presence of magnesium and chitin/carapace. The increase in gbpA gene expression is not hindered with increasing concentrations of magnesium irrespective of less number of bacterial colonies. Figure 3 shows a decline in the number of CFU/ml in all the three experimental setups supporting the literature regarding *V. parahaemolyticus* survival with increase in magnesium concentrations. Meanwhile the presence of carapace/commercial chitin also does effect the growth of the bacterium as observed clearly from the heat map too (Fig. 4).

**Table 1:** *Vibrio parahaemolyticus* mean colony forming units (CFU) per millilitre (ml) from the three experimental bacterial culture setups MgSO_4_·7H_2_O (NONE), MgSO_4_·7H_2_O with commercial chitin [CHITIN (0.05gm/3 ml)], and MgSO_4_·7H_2_O with carapace [CARAPACE (0.05gm/3 ml)]

Concentrations of MgSO_4_·7H_2_O	NONE (CFU/ml)	CHITIN (0.05gm/3 ml) (CFU/ml)	CARAPACE (0.05gm/3 ml) (CFU/ml)
0 ppm (0 mM)	148.850 × 10^3^	74.033 × 10^3^	137.95 × 10^3^
300 ppm (1 mM)	117.6 × 10^3^	43.33 × 10^3^	103.9 × 10^3^
18,500 ppm (75 mM)	87.8 × 10^3^	17.93 × 10^3^	90.7 × 10^3^
34,000 ppm (137 mM)	60 × 10^3^	15.96 × 10^3^	79.05 × 10^3^
55,500 ppm (225 mM)	57.9 × 10^3^	5.63 × 10^3^	24 × 10^3^
73,941 ppm (300 mM)	56.55 × 10^3^	3.7 × 10^3^	14.95 × 10^3^

The table shows colony-forming units at different MgSO_4_·7H_2_O concentrations (in ppm) of 0, 300, 18,500, 34,000, 55,000 and 73,941 ppm

## Conclusion

Our bioinformatics analysis carried out on the gbpA gene indicated likely interactions with chitin, an important component of the outer carapace of the prawn *M. rosenbergii*. The in vitro experiment on the gbpA expression of *V. parahaemolyticus,* with the *M. rosenbergii* components (mimicked commercial chitin and original prawn carapace) present in an external environment containing magnesium, meanwhile showed that gbpA gene expression was regulated. The results revealed comparatively equalized levels of gbpA gene expression in the absence of magnesium (0 mM) in the three experimental setups. On the other hand, significant changes in the gbpA gene expression were observed in the three experimental setups as explained under the results and discussion. All this points up to the importance of the environment containing magnesium in regulating the gene expression of bacterial gbpA.

The patterns of the gene expression of gbpA we observed could help increase our understanding of both the role of magnesium as the environment and of the host component chitin as a trigger for the pathogenic gene to respond. This work further provides pioneer information that the gbpA gene expression of *V. parahaemolyticus* pathogen may increase during the moulting phase of the prawn, when the mature prawn carapace is shed (with higher availability of epithelial chitin in the environment) and a new carapace emerges, initially as a thin chitinous layer. Due to higher gbpA expression at this stage the chances of the binding ability of gbpA to chitin could be higher. This hypothesis generated from our study can only be proved with further research targeting the moulting phase of prawn [29].

Our results give a new perspective to the importance of host-pathogen-environment experiments both for the aquatic industries and for microbiologists dealing with host-pathogen research. Further, our principle findings could provide a base for future research to use several other pathogen-related genes to investigate the interactions between gbpA and chitin (pathogen and host) with magnesium as an important component in the environment through proteomics research.

## Additional file

10.1186/s13099-016-0105-5 *Vibrio parahaemolyticus* PCV08-7 details for the three experimental setups MgSO_4_·7H_2_O, with MgSO_4_·7H_2_O and Chitin, with MgSO_4_·7H_2_O and carapace. **Figure S1**: *Vibrio parahaemolyticus* PCV08-7 culture plates for the three experimental setups MgSO_4_·7H_2_O (AppFigure1), with MgSO_4_·7H_2_O and Chitin (AppFigure2), with MgSO_4_·7H_2_O and carapace (AppFigure3). **Table S1**: RNA and cDNA concentrations for the three experimental setups MgSO_4_·7H_2_O (AppTable1), with MgSO_4_·7H_2_O and Chitin (AppTable2), with MgSO_4_·7H_2_O and carapace (AppTable3), **Table S2**: Analysis of variance for the significant values of the experimental setup MgSO_4_·7H_2_O, **Table S3**: Analysis of variance for the significant values of the experimental setup MgSO_4_·7H_2_O and Chitin, **Table S4**: Analysis of variance for the significant values of the experimental setup MgSO_4_·7H_2_O and carapace, **Table S5**: Student two tail paired t-test of equal variance across the three experimental setups MgSO_4_·7H_2_O, with MgSO_4_·7H_2_O and Chitin, with MgSO_4_·7H_2_O and carapace.

## Abbreviations

gbpA: *N*-acetyl glucosamine binding protein
MgSO_4_·7H_2_O: magnesium sulfate heptahydrate
ppm: parts per million
Mg^2+^: magnesium ion
ppt: parts per thousand
LB broth: Luria-bertani broth
deaD: RNA helicase
2^−ΔΔCT^ method: 2[-delta delta C(T)] method
VPPCV08-7: *Vibrio parahaemolyticus* PCV08-7
ml: millilitre
CFU: colony forming units

## References

[1] Barrow GI, Miller DC, Whitman W (1974). Genus 1 *Vibrio*. Bergey’s manual of systematic bacteriology.

[2] Colwell RR, West PA, Maneval D, Remmers EF, Elliot EL, Carlson NE, Colwell RR (1984). Ecology of pathogenic *vibrio*s in Chesapeake Bay. *Vibrios* in the environment.

[3] Jackson H, Fujino T, Sakaguchi G, Sakazaki R, Takeda Y (1974). Temperature relationships of *Vibrio* parahaemolyticus. International symposium of *Vibrio* parahaemolyticus.

[4] Barrow GI, Miller DC, Fujino T, Sakaguchi G, Sakazaki R, Takeda Y (1974). Growth studies on *Vibrio* parahaemolyticus in relation to pathogenicity. International symposium of *Vibrio* parahaemolyticus.

[5] Daniels NA, MacKinnon L, Bishop R, Altekruse S, Ray B, Hammond RM, Thompson S, Wilson S, Bean NH, Griffin PM, Slutsker L (2000). *Vibrio* parahaemolyticus infections in the United States, 1973–1998. J Infect Dis.

[6] Daniels NA, Ray B, Easton A, Marano N, Kahn E, McShan AL, Del Rosario L, Baldwin T, Kingsley MA, Puhr ND (2000). Emergence of a new *Vibrio* parahaemolyticus serotype in raw oysters: a prevention quandary. JAMA.

[7] Sanyal SC, Sen PC: Human volunteer study on the pathogenicity of *Vibrio parahaemolyticus.* In: International Symposium of *Vibrio parahaemolyticus*. International symposium of *Vibrio parahaemolyticus*. Tokyo: Saikon; 1974: 227–30.

[8] DePaola A, Kaysner CA, Bowers J, Cook DW (2000). Environmental investigations of *Vibrio parahaemolyticus* in oysters after outbreaks in Washington, Texas, and New York (1997 and 1998). Appl Environ Microbiol.

[9] Bhattacharya M, Roy SS, Biswas D, Kumar R (2000). Effect of Mg(2+) ion in protein secretion by magnesium-resistant strains of Pseudomonas aeruginosa and *Vibrio parahaemolyticus* isolated from the coastal water of Haldia port. FEMS Microbiol Lett.

[10] Heinis JJ, Beuchat LR, Boswell FC (1978). Antimetabolite sensitivity and magnesium uptake by thermally stressed *Vibrio parahaemolyticus*. Appl Environ Microbiol.

[11] Akio K, Teshima S, Sasaki M (1984). Requirements of the juvenile prawn for calcium, phosphorous, magnesium, potassium, copper, manganese and iron. Mem Fac Fish.

[12] Hangsapreurke K, Thamrongnawasawat T, Powtongsook S, Tabthipwon P, Lumubol P, Pratoomchat B (2008). Embryonic development, hatching, mineral consumption, and survival of Macrobrachium rosenbergii (de Man)reared in artificial seawater in closed recirculating water system at different levels of salinity. Mj Int J Sci Tech.

[13] Rejeki S (2003). Accumulation of aluminium in the tissue of giant fresh water prawn (*Macrobrachium rosenbergii* de Man) exposed to acidic water contaminated with aluminium salt. J Coast Dev.

[14] Hameed ASS, Rahaman KH, Alagan A, Yoganandhan K (2003). Antibiotic resistance in bacteria isolated from hatchery-reared larvae and post-larvae of Macrobrachium rosenbergii. Aquaculture.

[15] Clark JWH, Lynn JW (1977). A Mg ++ dependent cortical reaction in the eggs of Penaeid shrimp. J Exp Zool.

[16] Damrongphol P, Jaroensastraraks P, Poolsanguan B (2001). Effect of various medium compositions on survival and hatching rates of embryos of the giant freshwater prawn *Macrobrachium rosenbergii* cultured in vitro. Fish Sci.

[17] Jude BA, Martinez RM, Skorupski K, Taylor RK (2009). Levels of the secreted *Vibrio cholerae* attachment factor GbpA are modulated by quorum-sensing-induced proteolysis. J Bacteriol.

[18] Wong E, Vaaje-Kolstad G, Ghosh A, Hurtado-Guerrero R, Konarev PV, Ibrahim AF, Svergun DI, Eijsink VG, Chatterjee NS, van Aalten DM (2012). The *Vibrio cholerae* colonization factor GbpA possesses a modular structure that governs binding to different host surfaces. PLoS Pathog.

[19] Sasmal D, Guhathakurta B, Bhattacharya SK, Pal CR, Datta A (1996). Role of cell-associated *N*-acetyl-d-glucosamine specific haemagglutinin in the adhesion of *Vibrio cholerae* O1 to rabbit intestinal epithelial cells in vitro. FEMS Immunol Med Microbiol.

[20] Bhowmick R, Ghosal A, Das B, Koley H, Saha DR, Ganguly S, Nandy RK, Bhadra RK, Chatterjee NS (2008). Intestinal adherence of *Vibrio cholerae* involves a coordinated interaction between colonization factor GbpA and mucin. Infect Immun.

[21] Kirn TJ, Jude BA, Taylor RK (2005). A colonization factor links *Vibrio cholerae* environmental survival and human infection. Nature.

[22] Syed KA, Beyhan S, Correa N, Queen J, Liu J, Peng F, Satchell KJ, Yildiz F, Klose KE (2009). The *Vibrio cholerae* flagellar regulatory hierarchy controls expression of virulence factors. J Bacteriol.

[23] Almagro-Moreno S, Pruss K, Taylor RK (2015). Intestinal colonization dynamics of *Vibrio cholerae*. PLoS Pathog.

[24] Kuhn M, von Mering C, Campillos M, Jensen LJ, Bork P (2008). STITCH: interaction networks of chemicals and proteins. Nucleic Acids Res.

[25] Tiruvayipati S, Bhassu S, Kumar N, Baddam R, Shaik S, Gurindapalli AK, Thong KL, Ahmed N (2013). Genome anatomy of the gastrointestinal pathogen, *Vibrio parahaemolyticus* of crustacean origin. Gut Pathog.

[26] Burland TG (2000). DNASTAR’s lasergene sequence analysis software. Methods Mol Biol.

[27] Livak KJ, Schmittgen TD (2001). Analysis of relative gene expression data using real-time quantitative PCR and the 2[-Delta Delta C(T)] Method. Methods.

[28] Frederiksen RF, Paspaliari DK, Larsen T, Storgaard BG, Larsen MH, Ingmer H, Palcic MM, Leisner JJ (2013). Bacterial chitinases and chitin-binding proteins as virulence factors. Microbiology.

[29] Tiruvayipati S, Bhassu S (2016). Host, pathogen and the environment: the case of *Macrobrachium rosenbergii, Vibrio parahaemolyticus* and magnesium. Gut Pathog.

