# Supplementary material for: Host, pathogen and environment: a bacterial gbpA gene expression study in response to magnesium environment and presence of prawn carapace and commercial chitin
Source: Gut Pathog. 2016 May 26;8:23. doi: 10.1186/s13099-016-0105-5 (PMC4880808; doi:10.1186/s13099-016-0105-5)
Supplement: Supplementary file 1 — 10.1186/s13099-016-0105-5 Vibrio parahaemolyticus PCV08-7 details for the three experimental setups MgSO4·7H2O, with MgSO4·7H2O and Chitin, with MgSO4·7H2O and carapace. Figure S1: Vibrio parahaemolyticus PCV08-7 culture plates for the three experimental setups MgSO4·7H2O (AppFigure1), with MgSO4·7H2O and Chitin (AppFigure2), with MgSO4·7H2O and carapace (AppFigure3). Table S1: RNA and cDNA concentrations for the three experimental setups MgSO4·7H2O (AppTable1), with MgSO4·7H2O and Chitin (AppTable2), with MgSO4·7H2O and carapace (AppTable3), Table S2: Analysis of variance for the significant values of the experimental setup MgSO4·7H2O, Table S3: Analysis of variance for the significant values of the experimental setup MgSO4·7H2O and Chitin, Table S4: Analysis of variance for the significant values of the experimental setup MgSO4·7H2O and carapace, Table S5: Student two tail paired t-test of equal variance across the three experimental setups MgSO4·7H2O, with MgSO4·7H2O and Chitin, with MgSO4·7H2O and carapace. [file 13099_2016_105_MOESM1_ESM.doc]

**Figure S1:** *Vibrio parahaemolyticus* PCV08-7 bacterial plate count **AppFigure1:** MgSO_4_.7H_2_O only **AppFigure2:** MgSO_4_.7H_2_O with chitin (Sigma) **AppFigure3:** MgSO_4_.7H_2_O with carapace.


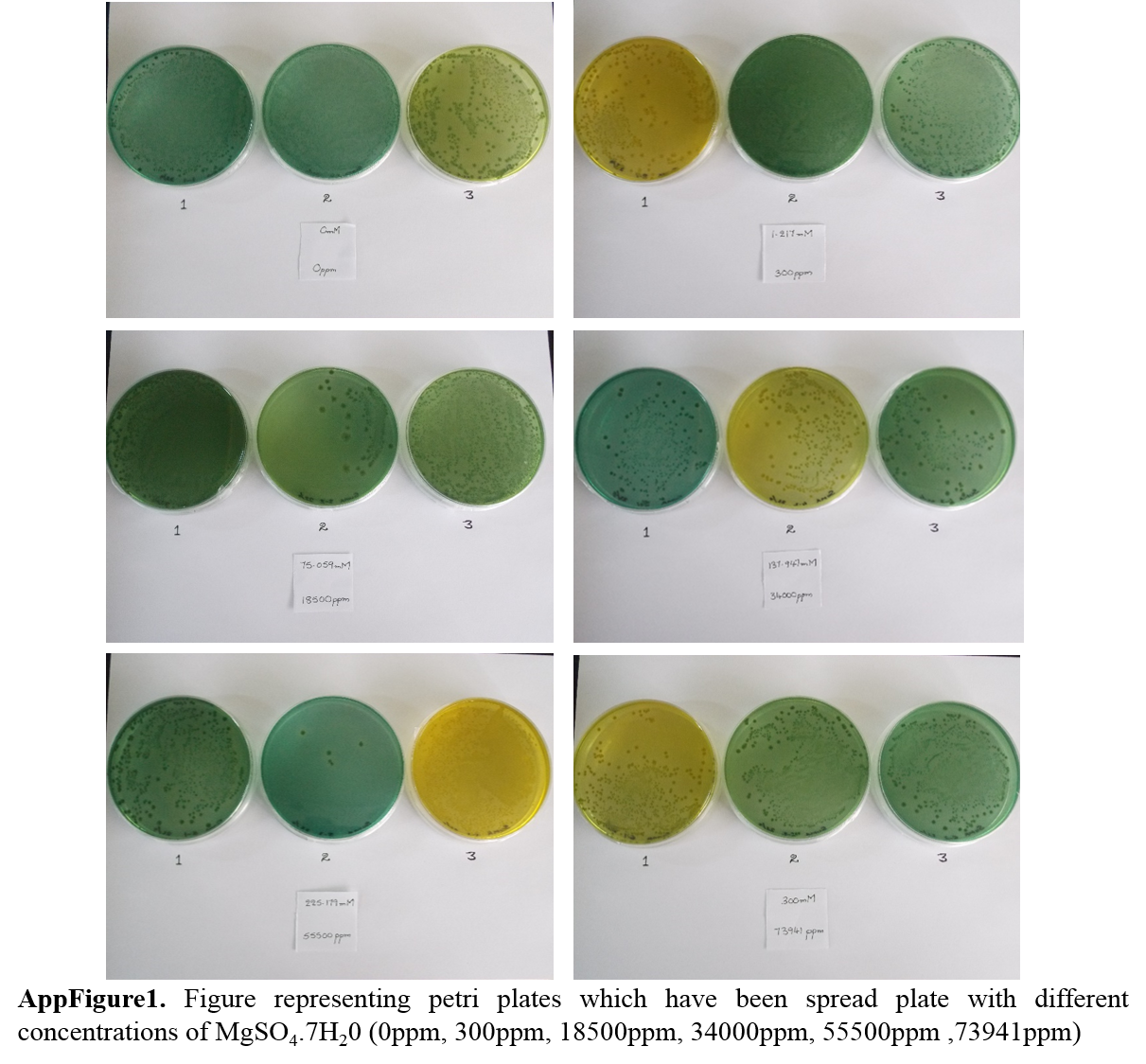


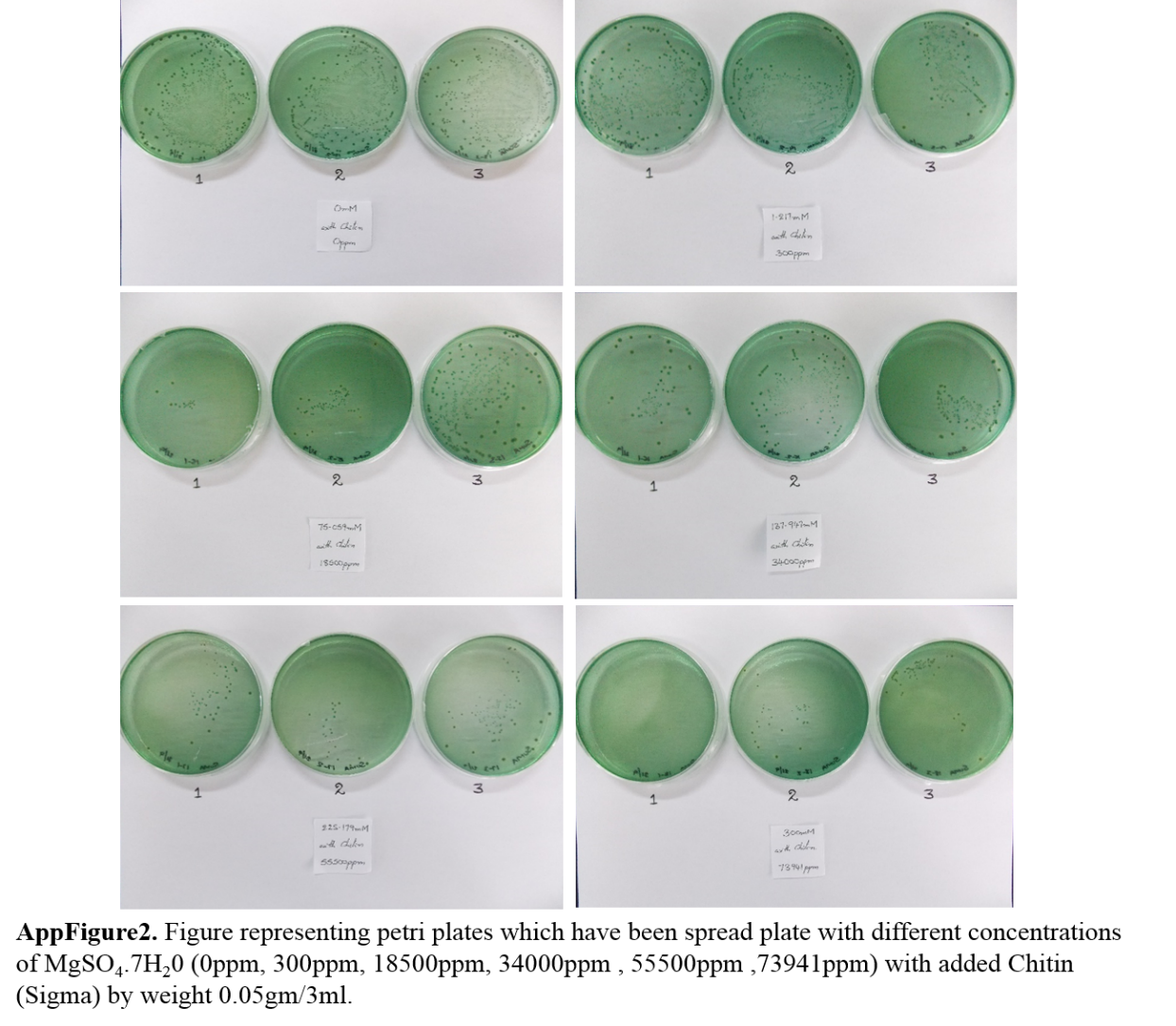


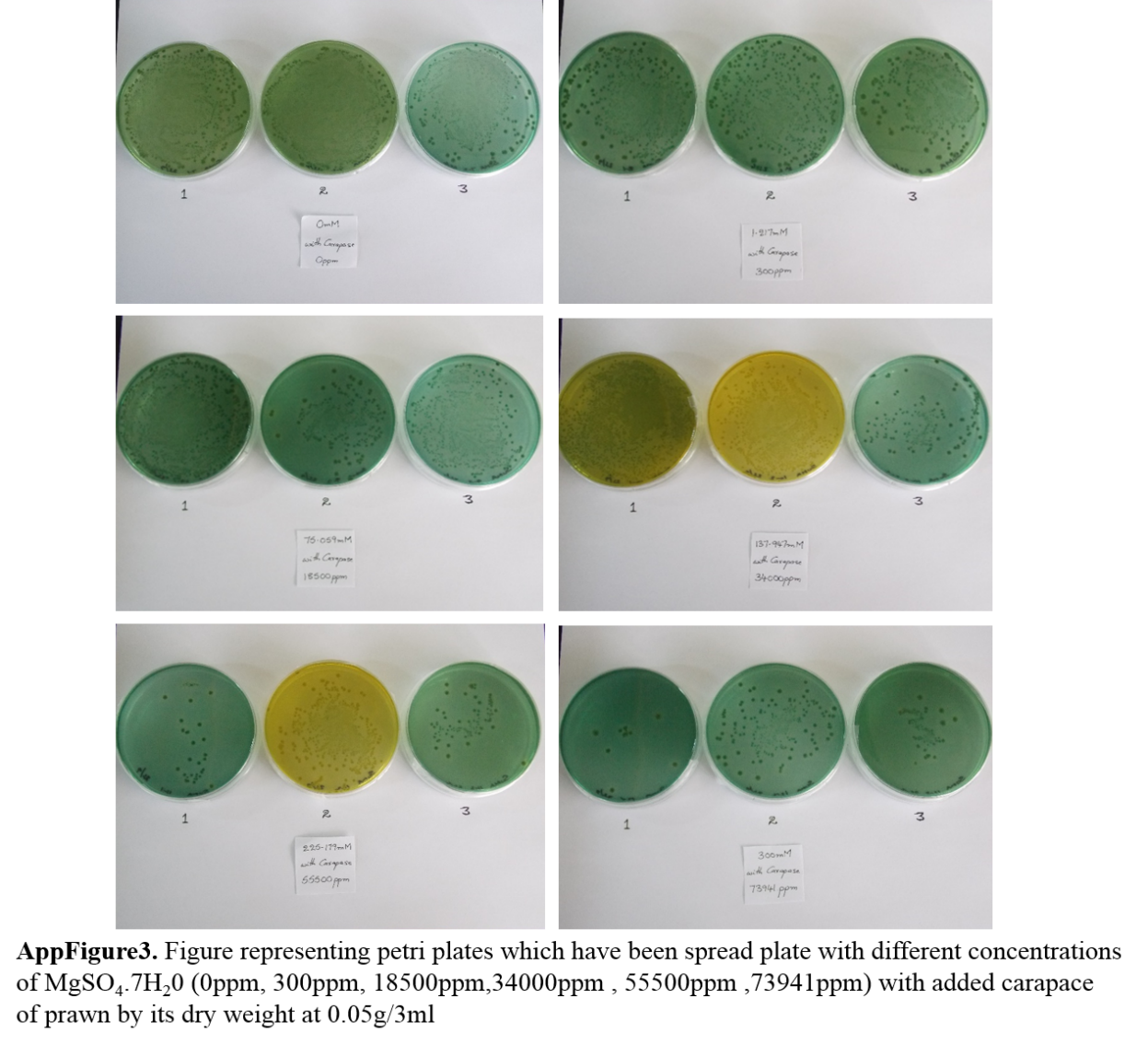


**Table S1:** *Vibrio parahaemolyticus* PCV08-7 RNA isolation and cDNA synthesis with Nanodrop quality readings

**AppTable1.** Table representing concentration of RNA and cDNA isolations for the different Magnesium concentration triplicates.


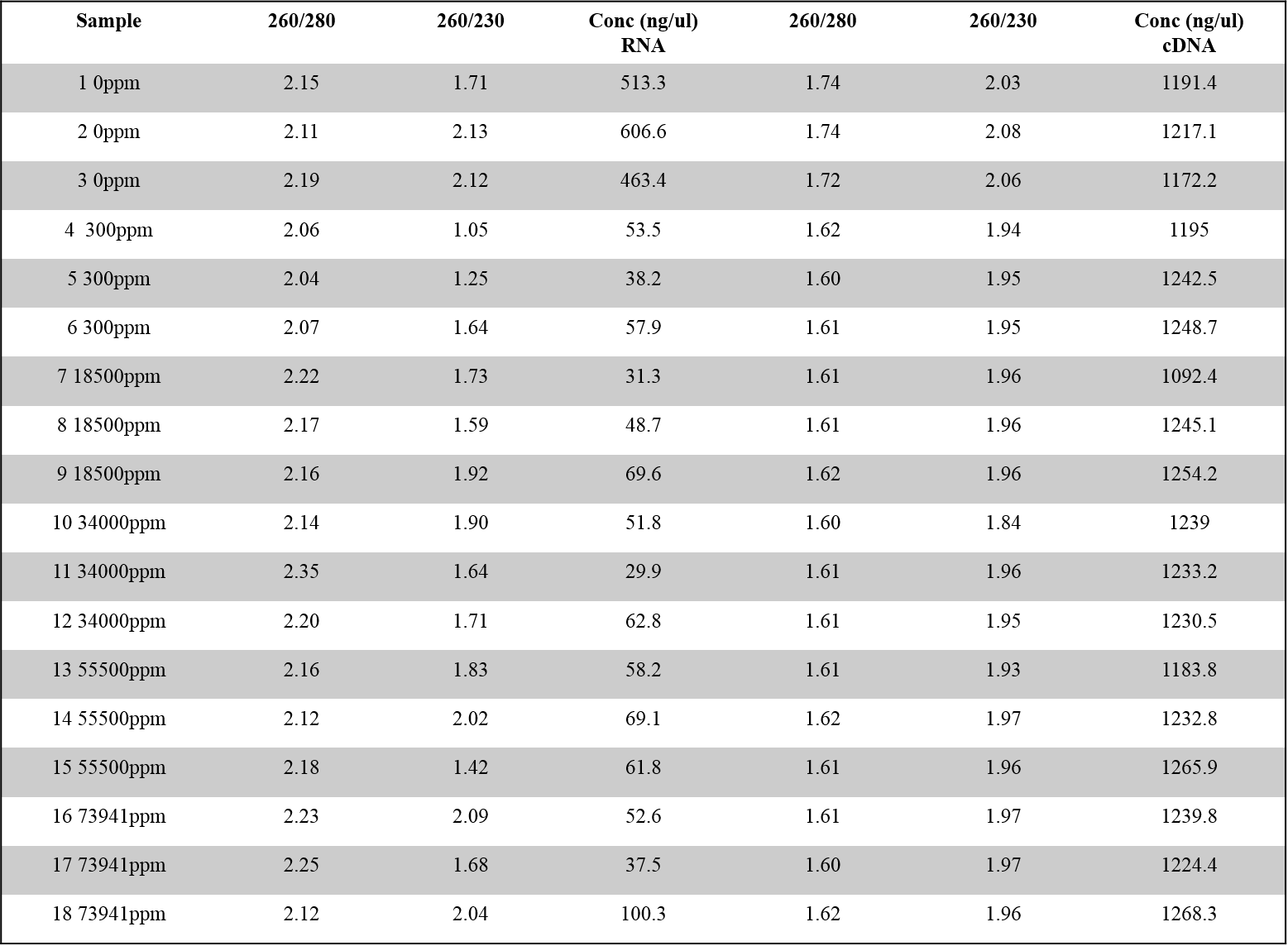


**AppTable2.** Table representing concentration of RNA and cDNA isolations for the different Magnesium concentration triplicates in the presence of Chitin.


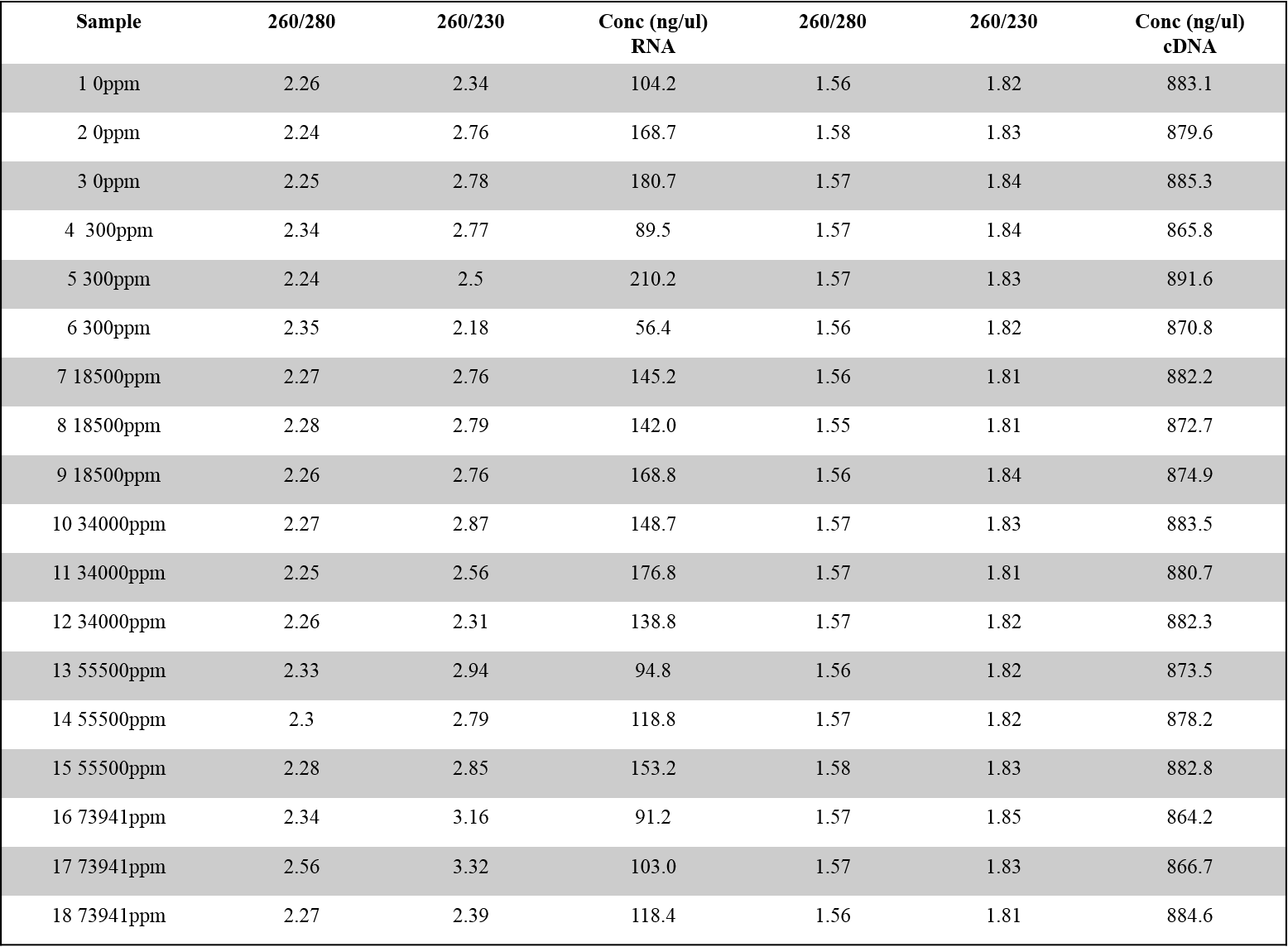


**AppTable3.** Table representing concentration of RNA and cDNA isolations for the different Magnesium concentration triplicates in the presence of carapace.


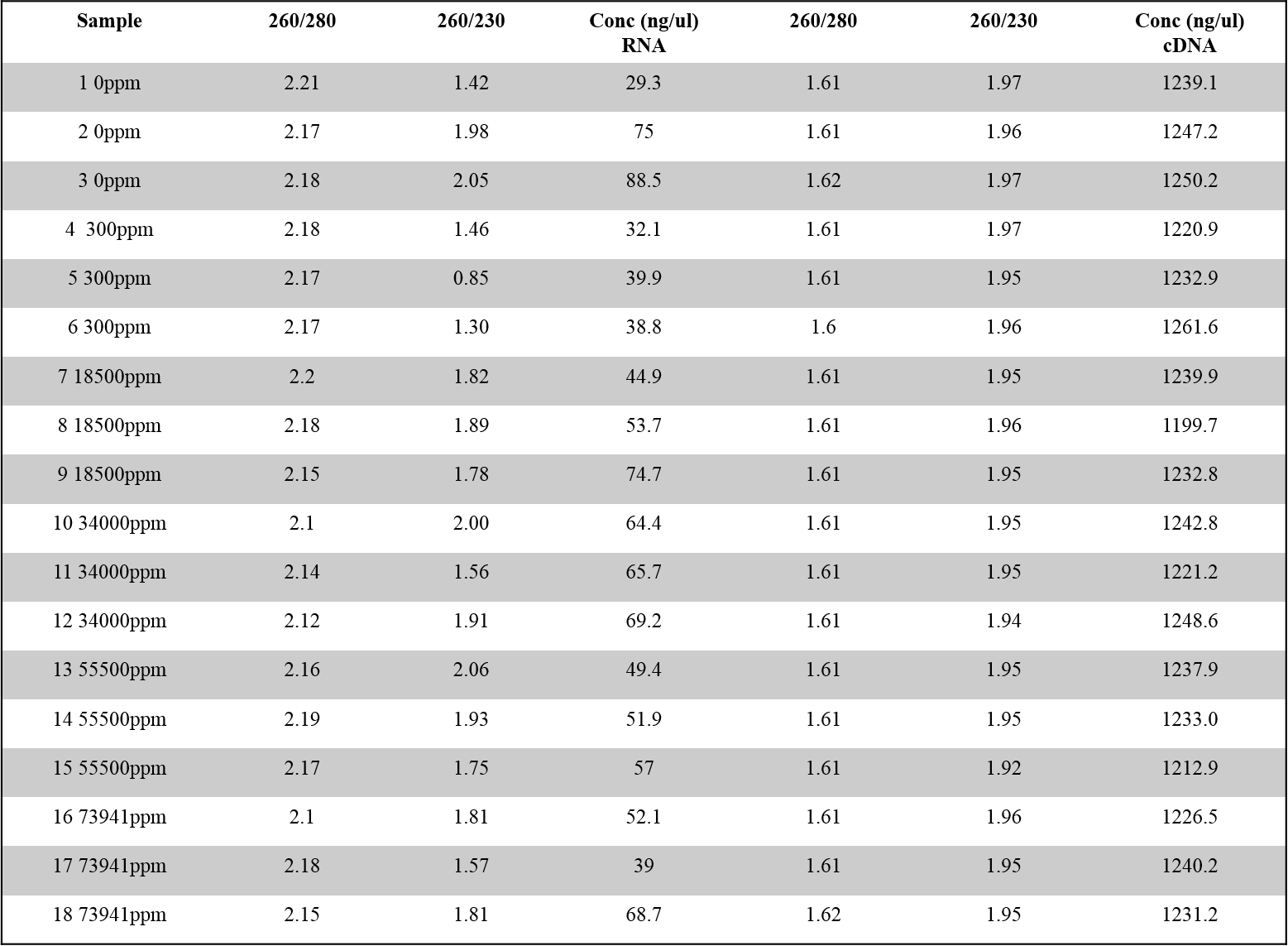


**Table S2:** Analysis of Variance to identify the level of significance for gene expression with the presence of MgSO_4_.7H_2_O

**
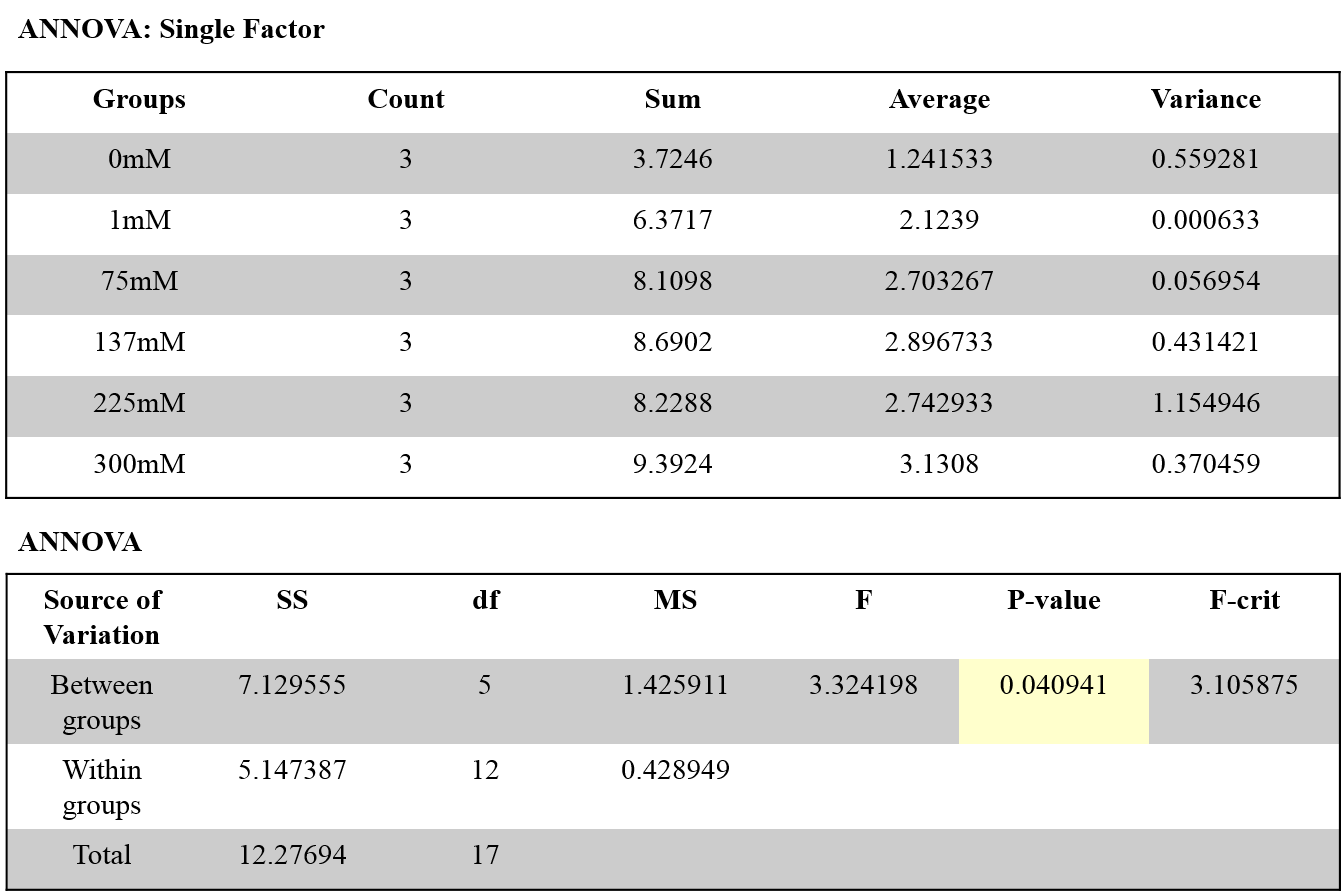
**

**Table S3:** Analysis of Variance to identify the level of significance for gene expression with the presence of MgSO_4_.7H_2_O and Chitin

**
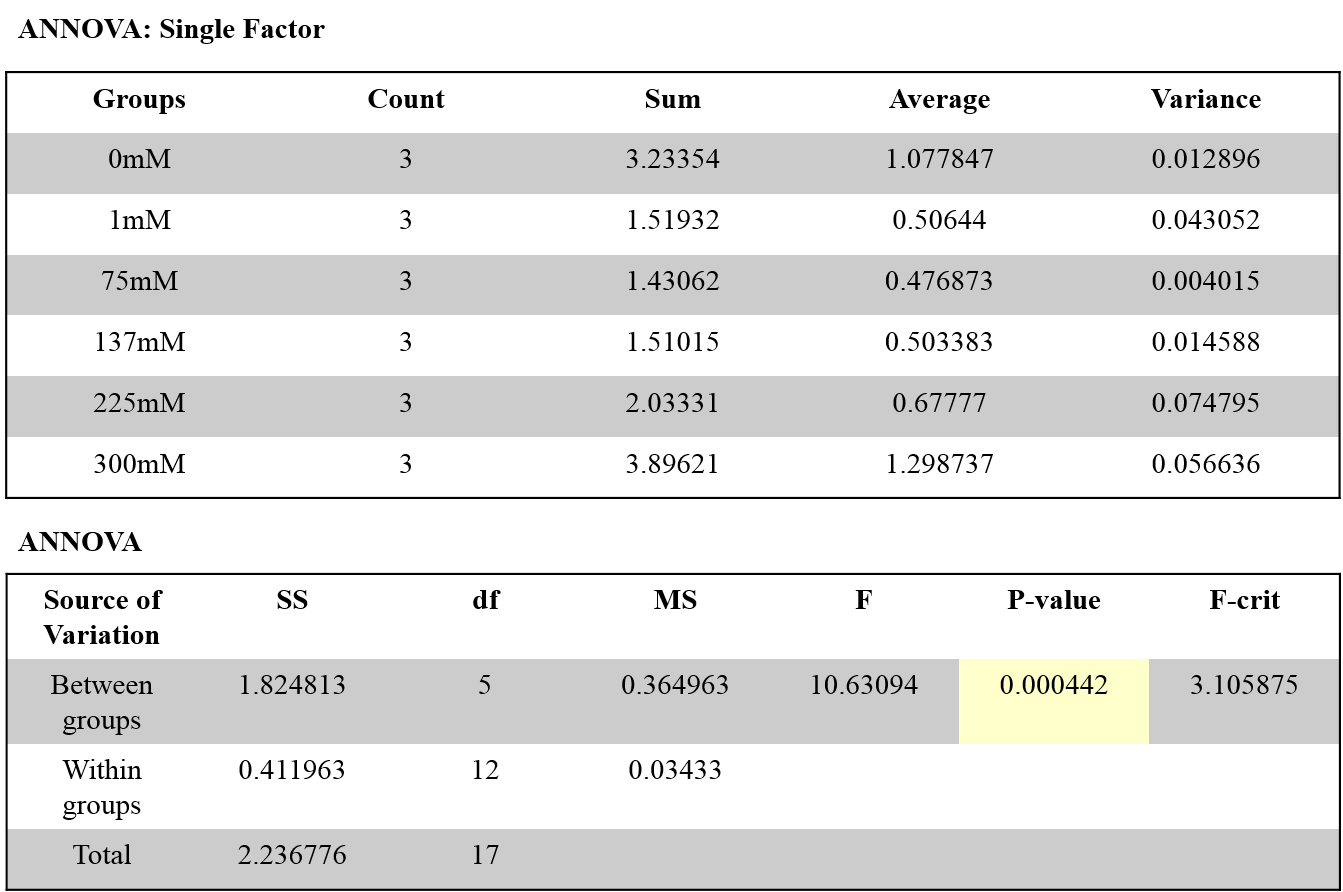
**

**Table S4:** Analysis of Variance to identify the level of significance for gene expression with the presence of MgSO_4_.7H_2_O and carapace

**
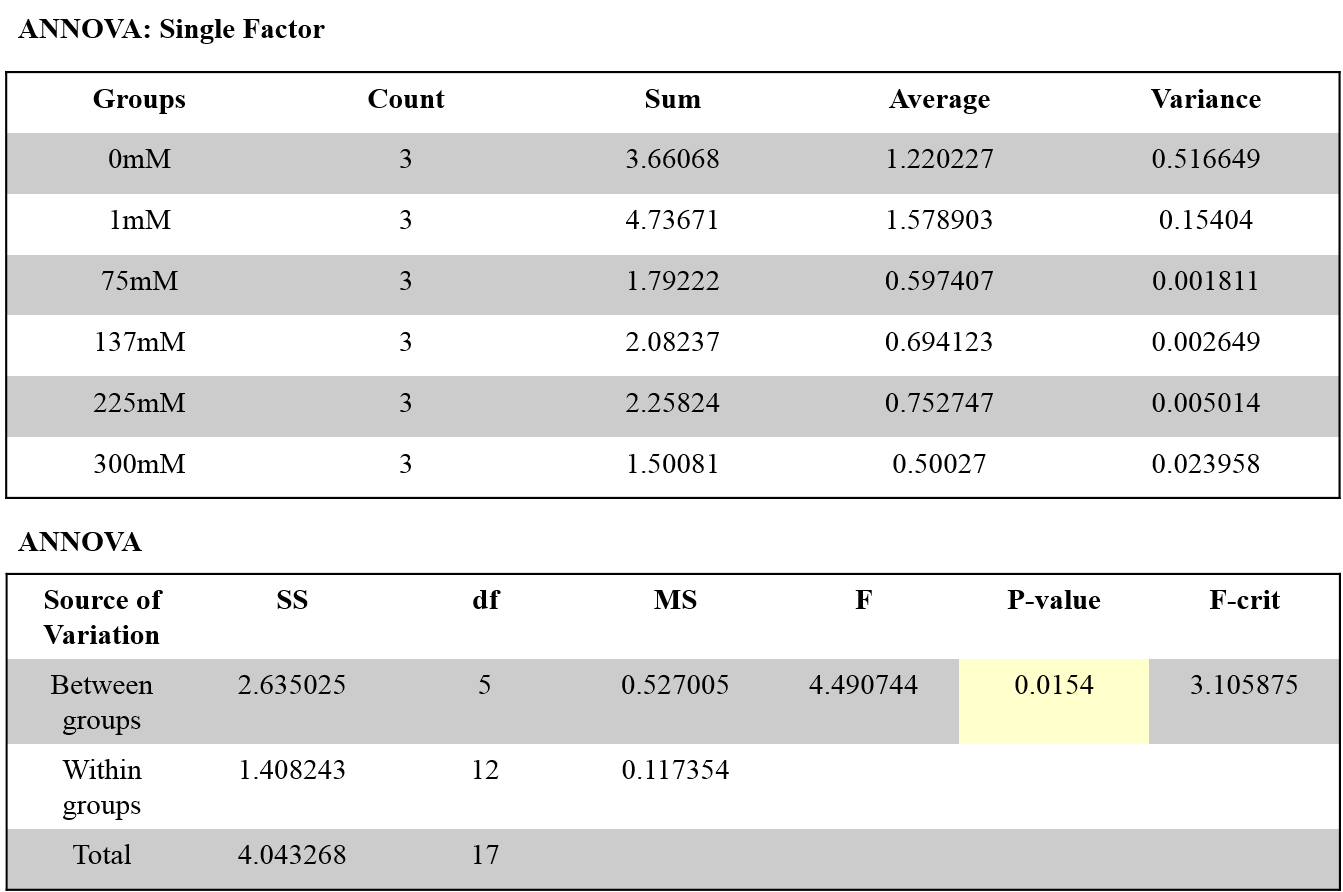
**

**Table S5:** Student two tail paired t-test of equal variance across gene expression samples of samples treated with only MgSO_4_.7H_2_O, with MgSO_4_.7H_2_O and Chitin, with MgSO_4_.7H_2_O and carapace

**
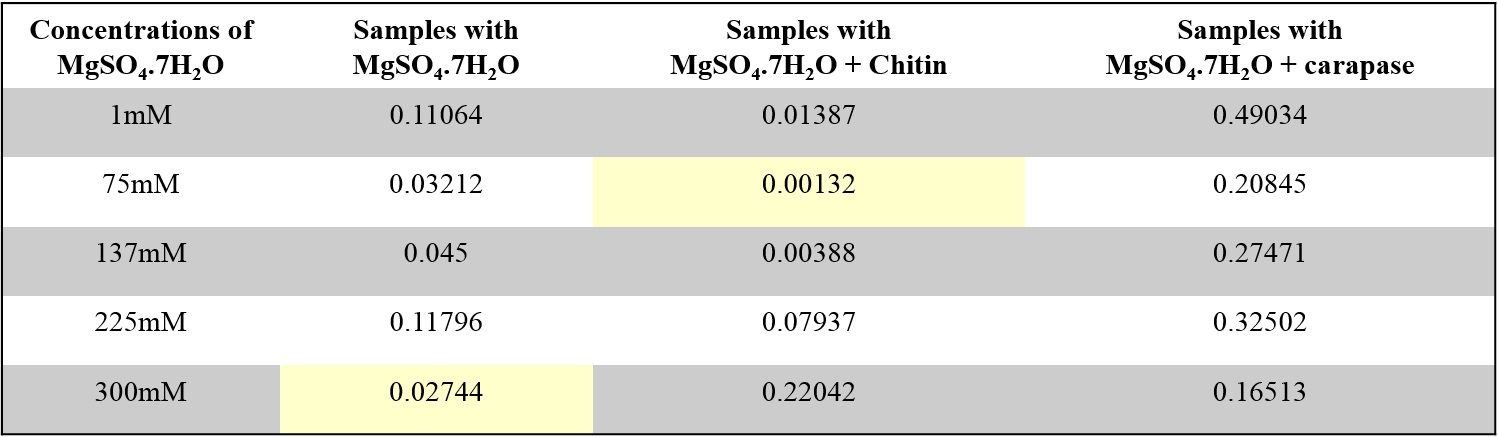
**
